# Editorial–data analysis in metabolomics

**DOI:** 10.1007/s11306-012-0418-4

**Published:** 2012-03-24

**Authors:** Jeroen J. Jansen, Johan A. Westerhuis

**Affiliations:** 1 Department of Analytical Chemistry, Institute for Molecules and Materials, Radboud University Nijmegen, Toernooiveld 1, 6525 ED Nijmegen, The Netherlands; 2 Biosystems Data Analysis, Swammerdam Institute for Life Sciences, University of Amsterdam, Science Park 904, 1098 XH Amsterdam, The Netherlands

The chemical diversity and the precision by which we are able to observe metabolism has taken a step change with the advent of metabolomics technology. Although the end goal of covering the entire metabolome by one chemical analysis is still far away, and indeed may never be realised considering the immense chemical diversity of metabolites, an ever-growing number of metabolites can be analysed in ever-lower concentrations. Also, analysis times become ever-shorter which dramatically increases throughput and therefore the number of samples a metabolomic study can contain.

To battle the resulting flood of data coming towards the biologist, researchers that employ metabolomics often turn to ‘chemometrics’, the research field that works on the representation of data from complex chemical analyses in simplified, interpretable models. The complexity of the biological and chemical background poses the chemometrician with a series of novel challenges. First of all, metabolomics is mostly used to gain insight in the biological system with highly specific underlying concepts while these standard methods provide a very generic view on the metabolism. Secondly, the latest developments in metabolomics also provide purely chemometric novel challenges: for example Nuclear Magnetic Resonance spectroscopy is used in metabolomics in a completely different fashion than in earlier applications, such as purity analysis in organic synthesis and protein structure elucidation. Furthermore, the metabolomic platforms themselves are pushing the limits of analytical chemical technology. Knowing, understanding and accounting for their (mis)behaviour in the large sample sets is also essential to make metabolomics a widely accepted systems-biological technology.

We have made it our task as guest editors to provide you with the newest developments in data analysis in metabolomics and to show to which lengths biological knowledge can be brought with this. All aforementioned aspects will be addressed in the manuscripts we selected for publication.

One of the drawbacks of data analysis is that it is generally highly technology-driven and therefore requires considerable programming skills. The contribution of Sun et al. describes their new toolbox COVAIN for many of the standard data analysis methods in current use for metabolomics, as well as many more advanced techniques. However, the toolbox brings them closer to the non-specialist researcher because it comes fitted with an easy-to-use graphical user interface. Also the contribution of Lei et al. covers new software: a new version of the package MET-IDEA for metabolomics data handling and processing. This program will considerably tighten the gap between the laboratory bench on which the mass spectrometer stands and the computer screen that depicts the data. The paper by Jankevics et al. is dedicated to this in silico screen and describes a method to determine the integrity and quality of the collected mass spectrometric data: applying this method should lead to more robust and information-dense metabolic profiles fit for subsequent data analysis.

Several other contributions focus on the models used for this data analysis, to provide results more focused upon the experimental factors relevant to the studied biological concepts. Koekemoer et al. show that one PCA model may not be able to cover all subtleties in different treatment groups and propose a method to model these and recover them in one large PCA model. The paper by Lemanska et al. shows how they were able pinpoint the effect oral rinse has on the metabolic composition of saliva with Analysis of Variance coupled to Simultaneous Component Analysis (ASCA), which is one of the methods developed in metabolomics to target a multivariate analysis more towards the biologically relevant information collected in the experiment. Xu and Goodacre explore the use of Consensus PCA to analyze bifactorial experimental data and show that it gives similar results to ANOVA-PCA while outperforming standard PCA analysis. A final challenge in multivariate analysis is to determine significance of experimental factors. For example in PLS-DA this can be done in several ways. Szymanska et al. compare several methods for this in their contribution and show which statistic measures are most powerful for model diagnostics. Finally, two contributions explore new signatures of metabolic variation to indicate system function. Jansen et al. propose to not only look at differences between treatment groups, but also at individual differences in metabolism and how they may be related to the experiment. The paper by Daykin et al. proposes to look beyond the small organic molecules freely dissolved in the biofluids, into the metabolites attached to macromolecules such as proteins. The specific NMR platforms used to collect the data to reveal such chemical interaction generate highly complex but well-structured data, which is subsequently used in the data analysis.

We feel the selected contributions not only show that data analysis is highly essential in all aspects of the metabolomics pipeline, but that this overview also shows that metabolomics is a truly interdisciplinary field in which the borders between biology, analytical chemistry and data analysis vanish more and more.

